# Histopathologic characterization of the BTBR mouse model of autistic-like behavior reveals selective changes in neurodevelopmental proteins and adult hippocampal neurogenesis

**DOI:** 10.1186/2040-2392-2-7

**Published:** 2011-05-16

**Authors:** Diane T Stephenson, Sharon M O'Neill, Sapna Narayan, Aadhya Tiwari, Elizabeth Arnold, Harry D Samaroo, Fu Du, Robert H Ring, Brian Campbell, Mathew Pletcher, Vidita A Vaidya, Daniel Morton

**Affiliations:** 1 Neuroscience Biology, Pfizer Global Research and Development, Eastern Point Road, Groton, CT 06340, USA; 2 Department of Biological Sciences, Tata Institute of Fundamental Research, Mumbai 400005 India; 3 FD Neurotechnologies, Inc, Ellicott City, MD 21041, USA; 4 Compound Safety Prediction Group, Pfizer Global Research & Development, Groton, CT 06340, USA; 5 Toxicologic Pathology, Pfizer Global Research and Development, 35 Cambridgepark Drive, Cambridge, MA 02140, USA

## Abstract

**Background:**

The inbred mouse strain BTBR T+ tf/J (BTBR) exhibits behavioral deficits that mimic the core deficits of autism. Neuroanatomically, the BTBR strain is also characterized by a complete absence of the corpus callosum. The goal of this study was to identify novel molecular and cellular changes in the BTBR mouse, focusing on neuronal, synaptic, glial and plasticity markers in the limbic system as a model for identifying putative molecular and cellular substrates associated with autistic behaviors.

**Methods:**

Forebrains of 8 to 10-week-old male BTBR and age-matched C57Bl/6J control mice were evaluated by immunohistochemistry using free-floating and paraffin embedded sections. Twenty antibodies directed against antigens specific to neurons, synapses and glia were used. Nissl, Timm and acetylcholinesterase (AchE) stains were performed to assess cytoarchitecture, mossy fibers and cholinergic fiber density, respectively. In the hippocampus, quantitative stereological estimates for the mitotic marker bromodeoxyuridine (BrdU) were performed to determine hippocampal progenitor proliferation, survival and differentiation, and brain-derived neurotrophic factor (BDNF) mRNA was quantified by *in situ *hybridization. Quantitative image analysis was performed for NG2, doublecortin (DCX), NeuroD, GAD67 and Poly-Sialic Acid Neural Cell Adhesion Molecule (PSA-NCAM).

**Results:**

In midline structures including the region of the absent corpus callosum of BTBR mice, the myelin markers 2',3'-cyclic nucleotide 3'-phosphodiesterase (CNPase) and myelin basic protein (MBP) were reduced, and the oligodendrocyte precursor NG2 was increased. MBP and CNPase were expressed in small ectopic white matter bundles within the cingulate cortex. Microglia and astrocytes showed no evidence of gliosis, yet orientations of glial fibers were altered in specific white-matter areas. In the hippocampus, evidence of reduced neurogenesis included significant reductions in the number of doublecortin, PSA-NCAM and NeuroD immunoreactive cells in the subgranular zone of the dentate gyrus, and a marked reduction in the number of 5-bromo-2'-deoxyuridine (BrdU) positive progenitors. Furthermore, a significant and profound reduction in BDNF mRNA was seen in the BTBR dentate gyrus. No significant differences were seen in the expression of AchE, mossy fiber synapses or immunoreactivities of microtubule-associated protein MAP2, parvalbumin and glutamate decarboxylase GAD65 or GAD67 isoforms.

**Conclusions:**

We documented modest and selective alterations in glia, neurons and synapses in BTBR forebrain, along with reduced neurogenesis in the adult hippocampus. Of all markers examined, the most distinctive changes were seen in the neurodevelopmental proteins NG2, PSA-NCAM, NeuroD and DCX. Our results are consistent with aberrant development of the nervous system in BTBR mice, and may reveal novel substrates to link callosal abnormalities and autistic behaviors. The changes that we observed in the BTBR mice suggest potential novel therapeutic strategies for intervention in autism spectrum disorders.

## Background

Autism is a behaviorally defined neurodevelopmental disorder consisting of impairment in reciprocal social interactions, deficits in communication, and restrictive and repetitive patterns of behaviors and interests. Preclinical and clinical advancement of novel therapies for the treatment of autism spectrum disorders (ASDs) would be greatly augmented by the existence of translational animal models for preclinical testing. Because the diagnostic and clinical outcomes of autism are based solely on behavioral outcome measures, neurobehavioral assessments have been a key focus of preclinical animal models. Several recent mouse models, generated using both forward and reverse genetic approaches, exhibit one or more behavioral deficits characteristic of autism [1-7].

It is widely acknowledged that the use of rodents to model complex neuropsychiatric disorders has significant limitations. Indeed, some neuroanatomic substrates that have been implicated in autism (for example, regions of the brain such as the fusiform gyrus [8] and cell types such as von Economo neurons [9]) are not present in rodents. However, in other complex central microtubule associated disorders, novel drug candidates can be tested in genetically defined mouse models to advance disease-modifying treatments. For example, genetically modified mice overexpressing amyloid plaques can be used to test novel drug candidates, despite the fact that these mice do not exhibit other cardinal features of human Alzheimer disease such as neurofibrillary tangles and neurodegeneration. With regard to autism, current human molecular genetics suggest that no single gene defines susceptibility to idiopathic autism [10]. Although several single-gene mouse models of monogenic forms of ASD exhibit behavioral deficits of autism [6,11], it is likely that no single-gene mouse model will be reliable for evaluating novel therapeutic candidates. Indeed, even in more homogenous single gene disorders such as fragile × syndrome, recent studies report heterogeneity in molecular signatures, which may translate to differential treatment responses [12].

An alternative and equally attractive approach is to employ both forward and reverse genetic mouse models in drug development for ASD. Mouse models of specific chromosomal aberrations represent a viable and appealing strategy for phenotypic characterizations in psychiatric diseases [4,13]. Inbred mouse strains can be powerful tools to decipher genes that confer susceptibility to distinct behavioral phenotypes. For example, forward genetics combined with quantitative trait loci mapping has defined specific loci that underlie a phenotype of behavioral despair [14,15]. Such approaches enhance confidence in the predictive validity of animal models for complex human diseases.

The BTBR inbred mouse recapitulates the three core behavioral features that define ASD, including deficits in social interactions as juveniles and adults, unusual vocalizations as infants, and repetitive stereotyped behaviors [3,6,16,17]. This strain is unique in that few mouse strains effectively model all three behavioral characteristics of autism [18]. More recent data in this model suggest that pharmacotherapy and behavioral intervention can reverse specific subdomains of the deficits [6,19-22]. One striking feature of the BTBR mouse is the complete absence of a corpus callosum. Agenesis of the corpus callosum in the BTBR model represents the most robust and fully penetrant callosal defect in any strain identified to date [23]. Other mouse strains display either callosal dysgenesis (partial reduction in callosal anatomy (for example, *Disc1 *truncation mice [24]) or less penetrant agenesis phenotypes (for example, in BALB/c mice, 10 to 18% of mice show callosal agenesis, and the incidence is gender-specific [25]). Because the callosal agenesis phenotype is fully penetrant in BTBR mice, this model represents a unique opportunity to explore the consequences of callosal abnormalities on brain structure and function. There is strong evidence that callosal abnormalities may be a crucial component associated with the core behavioral deficits of ASD. Human callosal abnormalities are rather common, with a reported frequency of 0.7 to 5.3% in the USA. Although many cases are asymptomatic, many patients born with callosal agenesis reportedly have cognitive and social impairments [26,27]. Despite the fact that most idiopathic cases of ASD do not show complete callosal agenesis, reductions in the volume of the corpus callosum is one of the most consistent neuroimaging finding in the brains of patients with autism [28-31]. A recent study showing that the corpus callosum undergoes changes in response to experience-dependent plasticity in the adult brain [32] suggests that alterations in white-matter microstructure can be modified by experience, and may be amenable to treatment. Such findings support mechanistic studies aimed at understanding the relationship between callosal abnormalities and ASD.

One approach to investigating the consequences of callosal defects is to characterize cellular and molecular changes in preclinical models, in which confounding factors such as postmortem delay and antemortem medications can be controlled. There is a paucity of reports describing the precise neuroanatomic, neurophysiological and neurochemical profile of the BTBR mouse. Neuroanatomic changes described to date in the BTBR strain are probably associated with absence of corpus callosum, and include the presence of Probst bundles [20], reduced hippocampal commissure [23] and an increased number of unmyelinated axons in the anterior commissure [33]. Functional changes reported in the BTBR model include augmented stress [34] and abnormal hippocampal neurophysiology [35,36].

We hypothesized that more complete histopathological characterization of the BTBR brain would reveal abnormal cellular and anatomic features that may correlate with the behavioral deficits and callosal abnormalities. In this study, we profiled a panel of neuronal, glial, synaptic and plasticity markers in the brains of BTBR and age-matched control mice. The markers were chosen to investigate neuropathologies that have been implicated in autism. The control inbred strain chosen for investigation, the B6 mouse, exhibits high levels of sociability, a low level of repetitive behaviors, and an intact corpus callosum [18,37]. Particular emphasis was placed on the forebrain limbic system, given the intimate relationship between the development of the corpus callosum and the limbic system, and the involvement of limbic system structures in ASD [38]. The relationship between behavioral deficits and neuropathologic findings in the BTBR model is discussed in terms of relevance to the translatability of the model to ASD.

## Methods

### Animals

All animals used in this study were housed in a facility accredited by the Association for Assessment and Accreditation of Laboratory Animal Care, International. All procedures related to animal care and treatment were conducted according to the guidelines of the Institutional Animal Care and Use Committee at Pfizer, the National Research Council Institute for Laboratory Animal Research *Guide for the Care and Use of Laboratory Animals*, and the US Department of Agriculture Animal Welfare Act and Animal Welfare Regulations.

Male BTBR mice and C57Bl/6J (B6) mice, 8 to 10 weeks old, were obtained from a colony maintained at the Jackson Laboratory (Bar Harbor, ME, USA). The age and gender were selected to match the conditions defined for behavioral phenotyping of the BTBR model in the literature [3], and in our own laboratory where we have replicated previously reported behavioral deficits [39]. Postmortem brain tissues were processed by free-floating techniques (n = 34 per strain), paraffin-wax embedding (n = 11 BTBR, n = 12 B6) and cryosectioning (*in situ *hybridization, n = 6 per strain). For histopathology, mice were anesthetized with a lethal dose of barbiturate (50 mg/kg Nembutal^®^; Abbott Laboratories, Abbott Park, IL, USA) and perfused transcardially with 0.1 mol/l phosphate buffer (pH 7.4), followed by 4% buffered paraformaldehyde. For *in situ *hybridization, whole frozen brains were excised from euthanized mice, and frozen in liquid nitrogen.

### BrdU treatment paradigm

BTBR and B6 mice received a single injection (200 mg/kg) of the mitotic marker BrdU (Sigma Aldrich., St Louis, MO, USA). To examine the proliferation of hippocampal progenitors within the adult hippocampal neurogenic niche, animals were euthanized 2 hours after BrdU administration (n = 9 per strain). To examine the long-term survival of newborn hippocampal progenitors, animals were euthanized 21 days after BrdU administration (n = 9 per strain). A schematic representation of the paradigm used for the study is shown (Figure 7A).

### Histology

#### Paraffin sections

Following perfusion, brains from BTBR and B6 mice were excised and post-fixed by immersion in fixative for 48 hours. Forebrains from six mice per strain were processed and embedded in the coronal plane in paraffin according to standard embedding techniques. Twenty serial sections, 5 μm in thickness, were collected from each of three coronal levels: prefrontal cortex (starting at Bregma 1.94 mm), striatum (starting at Bregma 0.98 mm) and dorsal hippocampus (starting at Bregma -1.70 mm). Anatomic landmarks were used to align coronal levels across the mice according to the atlas of Paxinos and Watson [40]. Whole brains from six mice per strain were used to prepared mossy-fiber blocks in the sagittal plane. Serial sections from sagittally embedded tissue were collected at the level of the dorsal hippocampus (starting at Bregma lateral 0.72 mm from the midline). Routine histopathology evaluation was performed on sections stained with hematoxylin and eosin (H&E).

Sectioning of all brains was carried out by attending closely to anatomic landmarks; special efforts were made to match levels between different mice, taking into account the distortion of key brain structures resulting from callosal agenesis. Brain regions implicated in the pathophysiology of autism were investigated, including the following high magnification regions: prefrontal cortex, corpus callosum, striatum, hippocampus and amygdala.

#### Free-floating sections

Brains from BTBR and B6 mice were prepared for cryosectioning to generate free-floating sections for immunohistochemistry and special stains. After perfusion, brains were excised and post-fixed by immersion in fixative for 6 hours at 4°C, and subsequently immersed in 0.1 mol/l phosphate buffer (PB) containing 20% sucrose for 72 hours. Tissue was rapidly frozen and stored at -75°C. Serial sections (30 μm thick) were cut coronally through the cerebrum, approximately from Bregma 2.96 mm to -4.96 mm [40]. Owing to the distortion of brain structures resulting from callosal agenesis in BTBR mice, different section intervals were used to match anatomic levels between the BTBR and B6 mouse brains. Thus, every first to tenth sections of each series of ten (for BTBR, interval = 300 μm) or twelve (for control, interval = 360 μm) sections were collected separately. The thickness of these sections facilitated visualization of structural elements that included processes such as secondary and tertiary dendrites of DCX positive neurons. One series of cryostat sections was processed for Nissl stain by staining sections with thionin, and evaluated qualitatively for gross neuroanatomic and cellular cytoarchitectural features.

#### Timm stain

Animals (n = 5 per strain) were deeply anesthetized (Nembutal; Abbott Laboratories) and perfused transcardially with Timm perfusate (FD Rapid TimmStain Kit™; FD Neurotechnologies, Baltimore, MD, USA), followed by 10% neutral buffered formalin. The brains were removed immediately, and post-fixed in the same fixative for 24 hours at 4°C. After cryoprotection in 0.1 mol/l PB containing 30% sucrose for 72 hours at 4°C, brains were then rapidly frozen and stored at -75°C. Serial cryostat sections 30 μm thick were cut coronally through the cerebrum, approximately from Bregma 2.80 mm to Bregma -4.36 mm [40]. Every first section of each series of five sections (interval 150 μm) was mounted on microscope slides (Superfrost Plus, VWR International, West Chester, PA, USA). After air-drying at room temperature for 2 hours, sections were processed for Timm staining (FD Rapid TimmStain Kit™; FD Neurotechnologies) according to the manufacturer's instructions http://www.fdneurotech.com. After two rinses in 0.1 mol/l phosphate buffer (pH 7.4), sections were incubated in the staining solution (made freshly by mixing solutions A to D from the kit) for 50 minutes at 30°C in the dark. Sections were dehydrated in graded ethanol, cleared in xylene, and coverslipped in mounting medium (Permount^®^; Fisher Scientific, Fair Lawn, NJ, USA).

#### Immunohistochemistry

##### Antibodies

Table 1 lists the antibodies used in the present study. After deparaffinization and rehydration, slides for immunohistochemistry requiring antigen retrieval were pretreated with a commercial antigen-retrieval system (Borg antigen retrieval; Biocare Medical, Concord, CA, USA), pH 9, using a pressure cooker. Slides of all levels from all animals were stained for a single antigen using an automated stainer (Autostainer Plus; Dako, Carpinteria, CA, USA). Endogenous peroxidase activity was blocked with 3% hydrogen peroxide prepared in 10% normal serum (Vector Laboratories, Burlingame, CA, USA). Primary antibodies were incubated for 2 hours and visualized with horseradish peroxidase (Envision HRP; Dako) using either rabbit or mouse secondary antibody conjugate. Immunoperoxidase reaction product was visualized with diaminobenzidine (DAB) chromagen (Dako). Paraffin- sections were counterstained using the Lillie modification of Mayer hematoxylin (Dako), and stained tissue sections were coverslipped with permanent mounting medium (Permount).

**Table 1:** Antibodies used for histopathological characterization of BTBR mice

*Antigen*	*Vendor*	*Catalog #*	*Host*	*Dilution*	*Retrieval*	*IHC Method*
**BrdU**	Accurate Chemical (Westbury, NY)	OBT0030	Rat IgG	1:300	None	Free-floating
	Roche Applied Science *(Indianapolis, IN)*	11170376001	Mouse IgG1	1:500	None	Free-floating
**CNPase**	Sigma-Aldrich *(St Louis, MO)*	C9743	Rabbit IgG	1:400	Borg	Paraffin
**Drebrin**	Sigma-Aldrich *(St Louis, MO)*	D3816	Rabbit IgG	1:1300	None	Paraffin
**Doublecortin**	Santa Cruz Biotechnology *(Santa Cruz, CA)*	sc-8066	Goat IgG	1:2000	None	Free-floating
**GAD65**	Millipore *(Temecula, CA)*	AB5082	Rabbit IgG	1:500	None	Paraffin
**GAD67**	Millipore *(Temecula, CA)*	MAB5406	Mouse IgG2a	1:1000 1:6000	Borg	Paraffin Free-floating
**GFAP**	Dako *(Carpinteria, CA)*	Z0334	Rabbit IgG	1:5000	Borg	Paraffin
	Sigma-Aldrich *(St Louis, MO)*	G9269	Rabbit IgG	1:300	None	Free-floating
						
**GluR1**	Millipore *(Temecula, CA)*	04-855	Rabbit IgG	1:50	Borg	Paraffin
**Iba1**	Wako Chemicals USA *(Richmond, VA)*	19-19741	Rabbit IgG	1:1000	Borg	Paraffin
**MAP2**	Sigma-Aldrich *(St Louis, MO)*	M4403	Mouse IgG1	1:1000	Borg	Paraffin
**MBP**	Abcam *(Cambridge, MA)*	ab2404	Rabbit IgG	1:50	Borg	Paraffin
**NeuroD**	Santa Cruz Biotechnology *(Santa Cruz, CA)*	sc-1086	Goat IgG	1:250	None	Free-floating
						
**NeuN**	Millipore *(Temecula, CA)*	MAB377	Mouse IgG1	1:1000	None	Free-floating
						
**NG2**	Millipore *(Temecula, CA)*	AB5320	Rabbit IgG	1:1200	None	Free-floating
**Parvalbumin**	EMD/Calbiochem *(Gibbstown, NJ)*	PC255L	Rabbit IgG	1:700	Borg	Paraffin
**PSA-NCAM**	Abcys *(Paris, France)*	AbC0019	Mouse IgM	1:5000	None	Free-floating
	Gift from Dr. T Seki	None	Mouse IgM	1:500	None	Free-floating
						
**PSD-95**	Cell Signaling Technology *(Beverly, MA)*	3409	Rabbit IgG	1:100	Borg	Paraffin
**Synapsin 2**	Sigma-Aldrich *(St Louis, MO)*	S2947	Rabbit IgG	1:1600 1:30,000	None	Paraffin Free-floating
**Synaptophysin**	Abcam *(Cambridge, MA)*	52636	Rabbit IgG	1:250	Borg	Paraffin
**VGluT1**	Synaptic Systems *(Goettingen, Germany)*	135303	Rabbit IgG	1:900 1:16,000	None	Paraffin Free-floating

IHC = immunohistochemistry; BrdU = 5-bromo-2'-deoxyuridine; CNPase = 2',3'-cyclic nucleotide 3'-phosphodiesterase; GAD65 = glutamic acid decarboxylase 65 kDa isform; GAD67 = glutamic acid decarboxylase 67 kDa isoform; GFAP = glial fibrillary acidic protein; GluR1 = glutamate receptor 1; Iba1 = ionized calcium-binding adaptor molecule 1; MAP2 = microtubule-associated protein 2; MBP = myelin basic protein;; NeuN = neuronal nuclei; PSA-NCAM = poly-sialic acid neural cell adhesion molecule; PSD-95 = postsynaptic density protein of 95 kDa; VGluT1 = vesicular glutamate transporter 1

For free-floating sections, sets of sections derived from BTBR and B6 mice were stained in series. The sections of the first set were mounted on slides and stained with thionin. The sections of the second set were processed for AchE histochemistry according to the method described by Naik [41]. The third to eighth sections of each set were stained with antibodies directed to selected antigens (Table 1). After inactivating the endogenous peroxidase activity with hydrogen peroxide, free-floating sections were incubated in 0.01 mol/l phosphate-buffered saline (PBS, pH 7.4) containing 1% normal blocking serum, 0.3% Triton X-100 (Sigma, St. Louis, MO, USA) and the primary antibody (Table 1). The immunoreaction product was visualized according to the avidin-biotin complex method (Vectastain elite ABC Kit; Vector Laboratories), followed by DAB. Stained sections were mounted on slides, dehydrated in ethanol, cleared in xylene and coverslipped (Permount; Fisher Scientific).

##### BrdU immunohistochemistry

BrdU immunohistochemistry was performed on every sixth hippocampal section. In brief, following DNA denaturation and acid hydrolysis, sections were treated with mouse anti-BrdU antibody and then exposed to biotinylated secondary antibody (Vector Laboratories). To further ascertain effects on adult hippocampal neurogenesis in BTBR mice, the numbers of immature neurons within the dentate gyrus that were immunopositive for DCX, PSA-NCAM and the NeuroD were examined. Brain coronal sections (six sections per animal) were treated overnight with DCX antibody, PSA-NCAM antibody or NeuroD antibody, followed by exposure to a biotinylated secondary antibody from a corresponding host species (Vector Laboratories). Signal amplification was carried out with an avidin-biotin complex (Vector Laboratories), and detected with DAB.

For triple-label immunofluorescence, sections subjected to DNA denaturation and acid hydrolysis were exposed to a mixture of antibodies (BrdU, NeuN)and glial fibrillary acidic protein (GFAP) overnight at room temperature. Following this, the sections were treated with a cocktail of secondary antibodies: biotinylated anti-rat (Vector Laboratories), Alexa-555 coupled donkey anti-mouse (Invitrogen, Carlsbad, CA, USA) and Cy5 coupled donkey anti-rabbit (Molecular Probes Inc., Eugene, OR, USA) for 2 hours, followed by incubation with fluorescein-conjugated streptavidin (Invitrogen) for 2 hours.

#### Antibody validation

Antibodies directed to various components of the nervous system were validated for immunohistochemistry in paraffin embedded and free floating coronal sections of B6 mouse brain. Validation included optimizing antibody conditions in naïve brains using antibody-titration experiments, antigen-retrieval techniques in paraffin and comparison of the observed staining pattern with published reports in the brain. Only those antibodies that had the expected distributions under conditions with optimal signal-to-noise detection were used for experimentation on the B6 and BTBR brain sections. A further confirmation of antibody specificity included determination that specific antibodies (GAD67, vesicular glutamate transporter VGluT1 and synapsin 2) had the same distribution in both paraffin embedded and free floating tissue (see Table 1).

#### *In situ *hybridization

Coronal sections 20 μm thick were cut on a cryostat (CM3050S; Leica Microsystems, Buffalo Grove, IL, USA) from frozen BTBR and B6 forebrains. Sections were staggered, so that each of the four sections on a slide was spaced 200 μm apart. Before use, the sections were immersed in 4% paraformaldehyde and then acetylated, defatted, and dehydrated through a graded series of alcohol.

Sense and antisense RNA probes specific for the 321 nucleotides between positions 507 and 833 in the NCBI database sequence M61178 of the BDNF mRNA sequence were used for *in situ *hybridization. A pCR3.1 plasmid (Invitrogen) was constructed with the antisense strand exposed by the restriction enzyme *Bam*H1, and transcribed by the S6 promoter. The plasmid with the sense strand used the linearization enzyme *Eco*RV, and the sequence was transcribed by the T7 polymerase. The riboprobes were labeled with ^35^S-dUTP (Perkin Elmer, Waltham, MA, USA) incorporation using a commercial kit (P6/T7 Riboprobe^® ^Combination System; Promega, Madison, WI, USA). The probe was then purified twice by ethanol precipitation.

Before hybridization, slides were incubated for 30 minutes in hybridization buffer containing 50% formamide, 2 × saline sodium citrate (SSC), 10% dextran sulfate, 1 × Denhardt solution, 1% denatured DNA, 0.01 mol/l dithiothreitol (DTT) and 10 μg/ml tRNA at 58 ºC. Slides were then incubated overnight at 58°C in hybridization buffer containing 2 × 10^6 ^counts per minute (cpm) sense or antisense probe per slide. Following hybridization, non-specific binding was removed by a series of stringency washes in decreasing salt concentrations at room temperature, ending with a stringency wash at 61°C and dehydration through a graded series of ethanol. After drying, the slides were exposed on an imaging plate (LSG TR Imaging Plate; Fuji Medical Systems, Stamford, CT, USA) for 14 days, and then scanned on a FLA7000 phosphorimager (Fuji Medical Systems).

### Microscopy

All stained slides were examined qualitatively using standard light microscopy. Whole-slide digital images of immunohistochemically stained slides were acquired using an imaging system (Aperio Scanscope XT; Aperio Technologies, Vista, CA, USA). Digital images were captured via a one-dimensional charge-coupled device line scan using a 40 × objective (0.75 numerical aperture, 0.25 μm/pixel) under constant exposure and light settings. Digital images were stored in the scanner's proprietary format (ScanScope Virtual Slide;.SVS) (compression quality = 70) and managed in the Aperio Spectrum database. High-magnification photomicrographs were taken with a 63 × oil immersion objective on a microscope (E-800; Nikon, Tokyo, Japan) with attached camera (SPOT RT; Spot Imaging Solutions, Sterling Heights, MI, USA).

### Image analysis

Quantification was carried out by an experimenter blinded to the study code.

#### Aperio image analysis

Quantitative image analysis was performed on sections in which there were qualitative changes in specific immunoreactivities in BTBR brains compared with B6 mouse brains. Additional selected markers that did not change were chosen as negative controls for direct comparison in the same animals. Quantification of NG2, DCX, PSA-NCAM and GAD67 immunoreactivities was performed using the software supplied with the imaging system (Aperio ImageScope; Aperio Technologies). This system is an automated whole-slide imaging system in which digital images are acquired and image analysis can be performed on stitched and tiled images analyzed at various magnifications; the system is being implemented with increasing frequency in quantitative studies of both rodent and human brain [42-45]. Acquired digital images representing whole tissue sections were analyzed applying the Spectrum Analysis algorithm package and ImageScope analysis software (version 9, Aperio Technologies). Quantification of immunohistochemical staining was performed using color translation and an automated thresholding algorithm. Percentage positivity per area traced was obtained using an algorithm that detects the chromogenic colorimetric reaction product. The area of immunoreactivity in each section was averaged to generate a mean immunoreactive value for each animal.

For NG2 quantification, image analysis was performed in the cingulate cortex and striatum. A rectangular region of interest (ROI) centered at the midline, 2.5 mm^2 ^in size, was defined using the cingulum as the medial-lateral landmarks and the pial surface as the dorsal boundary. In addition to the midline ROI, an additional rectangular ROI (0.8 mm^2^) centered in each hemisphere (right and left) of the striatum was quantified for NG2 immunoreactivity. Image analysis at the level of the dorsal hippocampus was performed for NG2, DCX, PSA-NCAM and GAD67. Using a freehand pen tool, an ROI was manually traced around the dentate gyrus to capture neurites in both the hilus and molecular/granule layers. Artifacts and white spaces were excluded by manual outlining using a negative pen tool. For all markers examined, images were manually uploaded from the Spectrum database and the positive-pixel-count macro was applied. A constant threshold was chosen, and all other parameters were held constant. The total area of specific stain in the defined ROI was assessed. Image analysis was performed using segmented stitched and tiled images (256 × 256 pixels). Data were manually exported to a spreadsheet (Excel; Microsoft Corp., Redmond, WA, USA) for data analysis. The final measurement consisted of the ratio of thresholded area of specific stain divided by the area of total ROI per section. The value for each animal consisted of a mean ratio for that animal, assessed in three equally spaced sections. For the striatum and hippocampus measurements, a mean value of the left and right hemispheres was calculated separately for each animal, and then averaged to yield a final mean value for each animal. Quantitative image analysis was not performed on H&E- or Nissl stained sections.

#### Unbiased stereology cell-counting analysis

Quantification of BrdU-positive cells in tissue sections was carried out using an unbiased design-based stereology protocol [46] on a microscope (AxioSkop; Zeiss, Jena, Germany) at a magnification of × 40. Tissue for this purpose was derived from free-floating sections. Every sixth section was taken, spanning the rostrocaudal extent of the hippocampus (nine sections/animal), and BrdUmmunopositive cells directly within or touching the subgranular zone (SGZ) or granule cell layer (GCL) were counted. The total number of BrdU-positive cells per SGZ or GCL was estimated by multiplying the total number of BrdU positive cells counted by section periodicity. To determine the number of cells in the hippocampus that were positive for DCX, NeuroD or PSA-NCAM, the cells in the DG were quantified, and results were expressed as the number of cells per section that were positive for each antibody. Furthermore, we also examined the morphological status of DCX positive cells by categorizing them as DCX positive cells with or without tertiary dendrites, as described previously [47]. To examine the differentiation of BrdU-positive cells into neurons positive for the neuronal marker NeuN, or into glia immunopositive for the glial marker GFAP, the percentages of BrdU positive cells that colocalized with NeuN or GFAP were assessed. A minimum of 30 BrdU positive cells from each animal was analyzed using z-plane sectioning with a confocal microscope (LSM5; Zeiss).

#### Densitometry

BDNF mRNA was quantified by measuring the densitometric expression level bilaterally on films from two to three sections per animal. The average signal value derived in this way was considered as n = 1 for statistical purposes. Regional analysis was performed with a freehand drawing tool (Multi Gauge software, version 3.2; Fuji Medical Systems). Criteria for selecting the CA1, CA2, CA3 and DG consisted of manually outlining regions based on matched morphological features from -1.5 to -2.5 mm Bregma [40]. The hippocampal regions from each section were normalized to a small region of lateral thalamus with nominal BDNF gene expression. A logarithmic regression of a ^35^S standard (American Radiolabeled Chemicals, St. Louis, MO, USA) included on each imaging plate was used to transform optical density values into nCi/g for cross plate standardization of data.

### Statistical analysis

Statistical analysis of quantitative immunohistochemistry image analysis data from the Aperio system was performed as follows: data for percentage stained area were log-transformed and then averaged for three sections. The averaged log-transformed data for individual animals were analyzed using a two sample *t*-test with equal variance to compare the values from the two strains of mice. The ratio of the two groups and its 95% confidence interval were also calculated. A mixed-effects model with strain and potential covariates as fixed effect and animal as random effect was used to quantitatively assess variability both between and within animals, as well as effect of strain and potential covariates.

For unbiased stereology cell counts (BrdU, DCX, PSA-NCAM and NeuroD immunohistochemistry and percentage colocalization of BrdU with NeuN and GFAP) data were subjected to statistical analysis using Student *t*-test with significance determined at *P *< 0.05 compared with the wild-type controls (GraphPad Instat software; GraphPad Software Inc., La Jolla, CA, USA).

For *in situ *hybridization densitometry, statistical significance was achieved at *P *< 0.05. All analyses were performed using SAS V9.2. Regions were compiled into groups and analyzed with a two-way ANOVA and a Bonferroni *post hoc *analysis (GraphPad Prism software)

## Results

### Gross Neuroanatomical differences exist between BTBR and B6 strains

**Figure 1:**
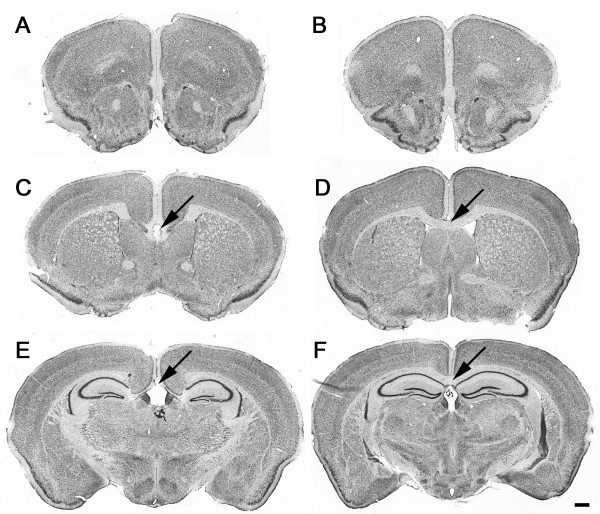
**Callosal agenesis is present in the BTBR mouse**. Nissl-stained free-floating coronal brain sections of **(A, C, E) **representative BTBR and **(B, D, F) **B6 mice at the level of **(A, B) **the prefrontal cortex, **(C, D) **striatum and **(E, F) **dorsal hippocampus. Gross anatomic findings include absence of corpus callosum (arrows) and consequential lateral displacement of the lateral septum, striatum and hippocampus. Scale bar = 500 μm.

Brains of BTBR mice were compared with age-matched B6 mice, using evaluations of gross morphology and routine histology. Evaluation of brains sectioned in both sagittal and coronal planes revealed significant lateral displacement of specific anatomic structures in the forebrain, such as the hippocampus, lateral septum and striatum (Figure 1). Such distortions were attributed to callosal agenesis, and have been described previously in this strain [23]. Ventricular dilation of the cerebral lateral and third ventricles was not seen in BTBR mice. The cerebellum and brainstem in the sagittal sections displayed no specific changes in BTBR versus controls at the gross or cellular level (data not shown). No significant change in total brain weights was seen in BTBR versus B6 mice at 8 to 10 weeks of age (BTBR: 0.55 ± 0.06 g; B6: 0.57 ± 0.06 g), which is consistent with previous observations of the BTBR versus B6 strains evaluated at 12 weeks of age [23].

Regions for analysis in the present study were based on brain regions implicated in the neuropathology of autism [38] and the established callosal connectivity of forebrain structures. The following regions were evaluated: isocortex, hippocampus, amygdala, septum and striatum. In the prefrontal cortex, there were no obvious anatomic or morphologic differences between BTBR and B6 mice. Interhemispheric white-matter abnormalities at the midline were apparent in both striatal and hippocampal coronal levels. The corpus callosum was consistently absent in all BTBR mice examined. The external capsule likewise appeared reduced in thickness in BTBR mice compared with controls, whereas the thickness of the internal capsule and anterior commissure appeared indistinguishable from controls, suggesting anatomic specificity in white-matter tracts. The size of the striatum and septum, thickness of the cortex and size of the ventricles were not noticeably different in BTBR and B6 mice. At the level of the dorsal hippocampus, lateral displacement of the hippocampus was apparent in all BTBR mice. The hippocampal commissure was reduced in size, consistent with previous reports [23]. Specific anatomic changes in BTBR hippocampus included an apparent reduction in the thickness of the hippocampal dentate granule neuron layer as well as thinning of the hilus (Figure 1E, Figure 7; see Additional file 1, Figure S3). No obvious changes in the anatomic structure or cytoarchitecture of the amygdala were seen. At the cellular level, no evidence of neurodegeneration or gliosis was seen in H&E-stained or Nissl-stained sections in any brain area.

### Misorientation of selected glial fibers is present in the BTBR forebrain

**Figure 2:**
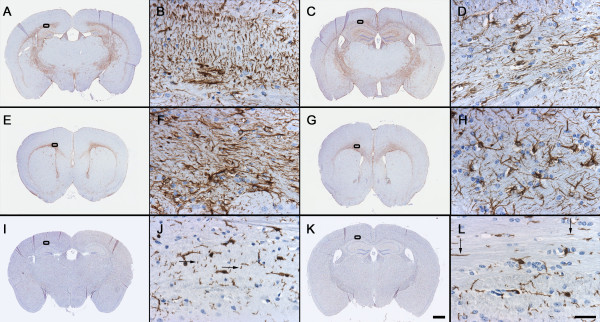
**Astrocytes and microglia show altered orientation in specific white-matter tracts in BTBR forebrain**. Low-magnification coronal sections from representative **(A, E, I) **BTBR and **(C, G, K) **B6 mouse forebrain at the level at which misoriented glial fibers were visible. Gross anatomic changes included lateral displacement of the **(A, I) **hippocampus and **(E) **lateral septum. Rectangular area illustrates region highlighted at high magnification in adjacent panels: **(A-D) **glial fibrillary acidic protein (GFAP) immunostaining of a representative BTBR versus B6 mouse illustrated at high magnification in **(B, D) **the alveus at the level of the dorsal hippocampus. The orientation of GFAP-immunoreactive fibers in the alveus seemed prominently directed in the dorsolateral axis in BTBR brain. **(E-H) **GFAP staining in the cingulum of **(F) **BTBR versus **(H) **B6 mouse forebrain at the level of the striatum. The orientation of GFAP-immunoreactive fibers in the cingulum seemed prominently directed in the mediolateral direction in **(F) **BTBR compared with **(H) **B6 brain. GFAP-immunoreactive fiber density seemed to be increased in BTBR white matter in tracks neighboring the absent corpus callosum (compare B, F with D, H). **(I-L) **Ionized calcium-binding adaptor molecule (Iba)1 immunoreactivity in the alveus of **(J) **the BTBR versus **(L) **the B6 mouse. GFAP and Iba1 positive fibers were oriented dorsoventrally in **(B, J) **the BTBR brain rather than mediolaterally as seen in **(D, L) **the B6 brain. **(J, L) **Arrows are perpendicular to the directionality of Iba1-positive fibers. GFAP- and Iba1-immunoreactive cell bodies seemed qualitatively indistinguishable in terms of size, frequency and morphology between BTBR and B6 mice. Scale bars = **(A, C, E, G, I, K) **1000 μm; **(B, D, F, H, J, L) **25 μm.

To more fully characterize glial phenotypes, localization of astrocytes and microglia was examined using GFAP and ionized calcium-binding adaptor molecule Iba1 [48] [AU immunohistochemistry, respectively. Figure 2 illustrates astrocyte and microglial expression in defined white-matter regions of the forebrain, namely the cingulum and the alveus. GFAP immunoreactive fibers did display some alterations specific to BTBR in selected regions. The density of GFAP-immunoreactive fibers was increased in the cingulum of the BTBR forebrain compared with that of B6 mice (Figure 2). Astrocytic processes were often oriented dorsoventrally rather than mediolaterally in the cingulum and alveus at the levels of the striatum and hippocampus (Figure 2). Likewise, Iba1-positive microglial fibers were oriented dorsoventrally in the alveus of BTBR mice (Figure 2). Misoriented glial fibers were confined to the white matter of these selected neuroanatomic regions, and were not seen in the gray matter. No evidence of cellular hypertrophy or hyperplasia was seen in GFAP or Iba1-immunoreactive cells. In the gray matter, morphologic cellular features of GFAP and Iba1-positive cells were indistinguishable in BTBR compared with B6 forebrain, consistent with a lack of astrogliosis or microgliosis. The misorientation of glial processes was only found in brain regions that normally receive corpus callosum innervations, indicating that these findings are likely to be a consequence of callosal agenesis.

### White-matter microstructure and NG2 oligodendrocyte precursors are altered in BTBR forebrain

**Figure 3:**
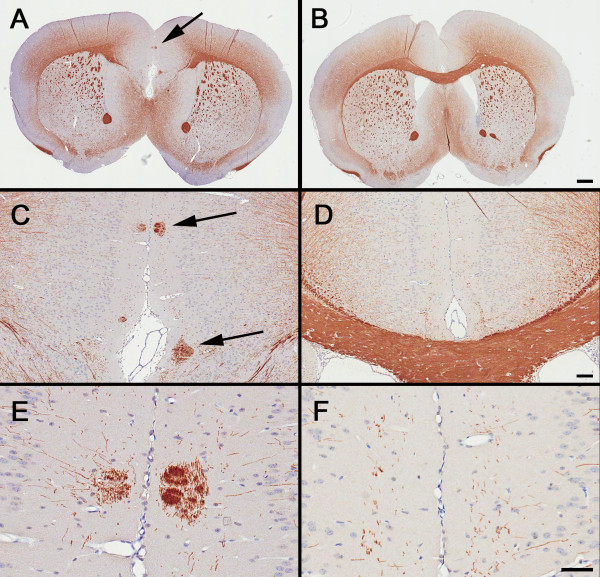
**Ectopic white-matter bundles expressing myelin basic protein (MBP) immunoreactivity are present in the anterior cingulate cortex (ACC) of the BTBR mouse**. Immunostaining of MBP in representative **(A, C, E) **BTBR and **(B, D, F) **B6 brains. **(A, B) **Absence of the corpus callosum was prominently seen at low magnification in BTBR. **(C, D)**. At higher magnifications, small ectopic bundles of MBP-immunoreactive white-matter tracts were seen in the ACC of BTBR brain (arrows). **(E, F) **High magnification of MBP immunoreactivity in the cingulate cortex. Scale bars = **(A, B) **500 μm, **(C, D) **100 μm, **(E, F) **50 μm.

**Figure 4:**
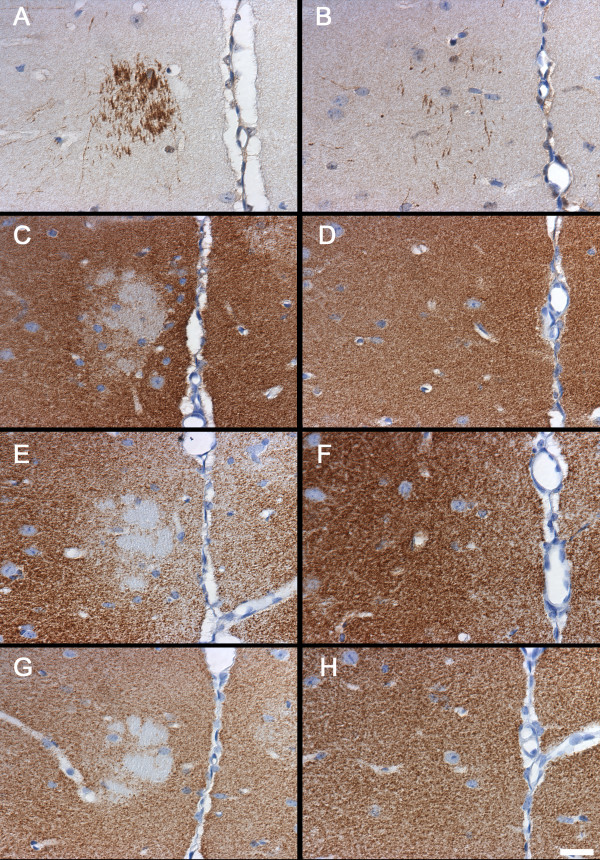
**Ectopic white-matter bundles in the anterior cingulate cortex (ACC) express 2',3'-cyclic nucleotide 3'-phosphodiesterase (CNPase) and lack synaptic antigens**. Immunostaining of CNPase and synaptic markers in the ACC of representative **(A, C, E, G) **BTBR and **(B, D, F, H) **B6 brains. The images are nearly adjacent to those shown in Figure 3. **(A) **Focal white-matter bundles had strong immunoreactivity for CNPase, a marker of oligodendrocytes and myelinated fibers. An absence of markers of pre and postsynaptic antigens, as revealed by **(C) **drebrin **(E) **and vesicular glutamate transporter VGluT1 and **(G) **synaptophysin immunoreactivities, was seen in the anterior cingulate cortex of BTBR brain. **(D, F, H) **Staining with synaptic markers appeared to be uniform in the corresponding cortical region from B6 mice. Scale bar = 25 μm.

Assessment of myelin was evaluated by immunohistochemical localization of myelin basic protein (MBP) and 2',3'-cyclic nucleotide 3'-phosphodiesterase (CNPase). Both antigens are expressed in myelinated fiber tracks; however, CNPase is also expressed in oligodendrocyte cell bodies [49]. Alterations included reduced expression of MBP and CNPase in the midline of the BTBR forebrain [at the level of the striatum (Figure 3; Figure 4) and hippocampus, findings that are consistent with callosal agenesis. MBP and CNPase immunoreactivities were aberrantly expressed in small, ectopic white-matter bundles located in the cingulate cortex adjacent to the midline (Figure 3; Figure 4). These bundles extended anterior to posterior throughout a relatively long longitudinal axis (Bregma 2.2 to -2.06). Typically, these white-matter structures ranged in size from 25 to 200 μm in diameter, and there were one to four foci per hemisphere. All BTBR mice examined had these ectopic white-matter bundles, which were not seen in any of the B6 mice.

**Figure 5:**
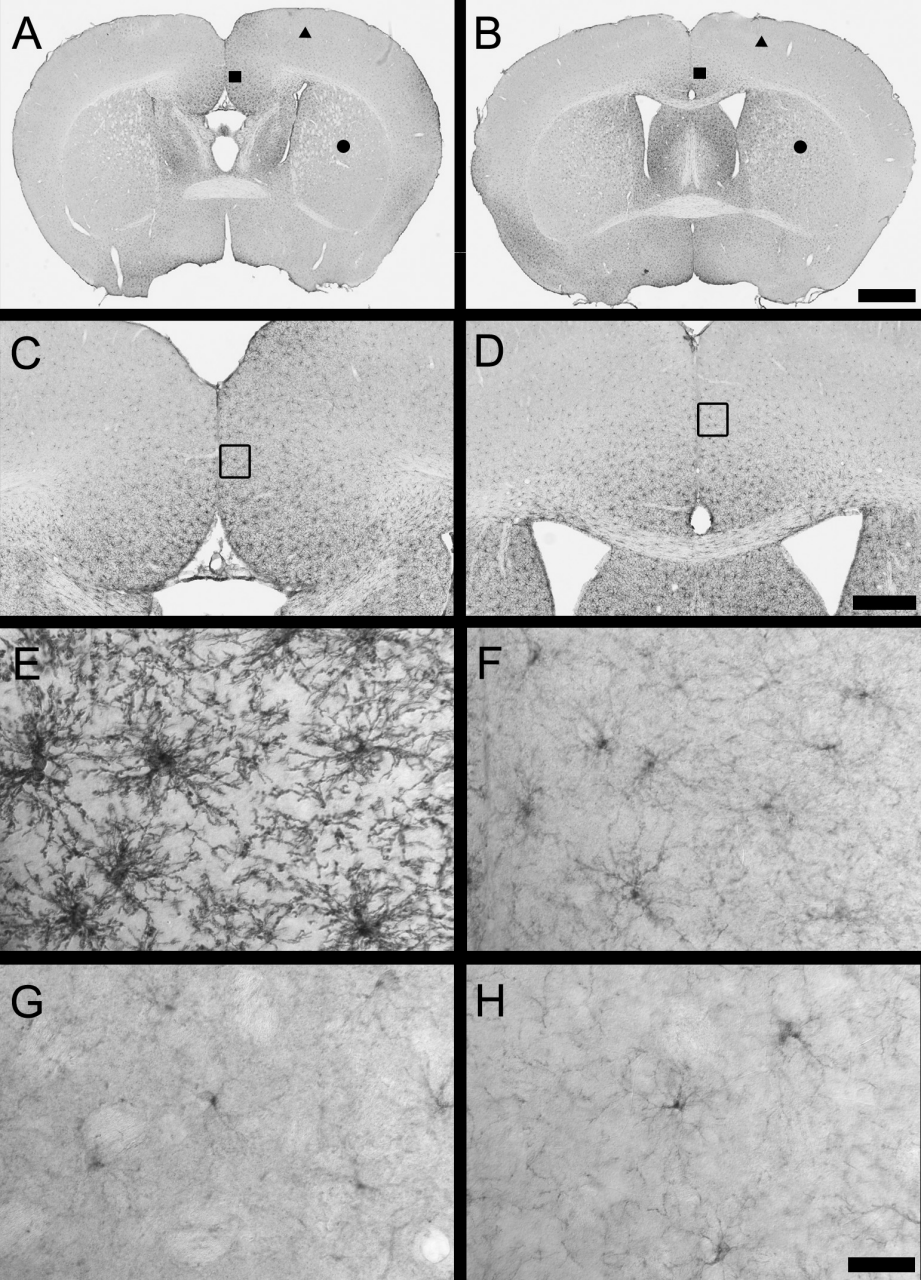
**Oligodendrocyte precursors defined by neuroglial proteoglycan NG2 immunoreactivity are qualitatively increased in the anterior cingulate cortex of the BTBR mouse**. Immunoperoxidase localization of NG2 in the forebrain of representative **(A, C, E, G) **BTBR and **(B, D, F, H) **B6 mice, at **(A, B) **low, **(C, D) **medium and **(E, F, G, H) **high magnifications. **(B) **Prominent cellular immunoreactivity of NG2 was visible throughout the brain of B6 mice with variation in expression levels in different brain regions. For example, constitutive levels of NG2 immunoreactivity in B6 mouse brain were higher in the septum and anterior cingulate cortex (square) than in the isocortex (triangle) or striatum (circle). **(C, D) **Increased magnification of (A, B) in the midline cortical region, illustrating NG2 immunoreactivity. **(E, F) **High-magnification illustration of increased NG2 staining in the ACC of the BTBR compared with the B6 mouse (from square region in (A, B) and open rectangle in (C, D), respectively). **(G, H) **High magnificationof NG2 staining in the BTBR and B6 mouse striatum, showing no qualitative difference in NG2 immunoreactivity (from circle region in (A, B)). Scale bars = **(A, B) **1000 μm, **(C, D) **500 μm, **(E, F, G, H) **25 μm.

**Figure 6:**
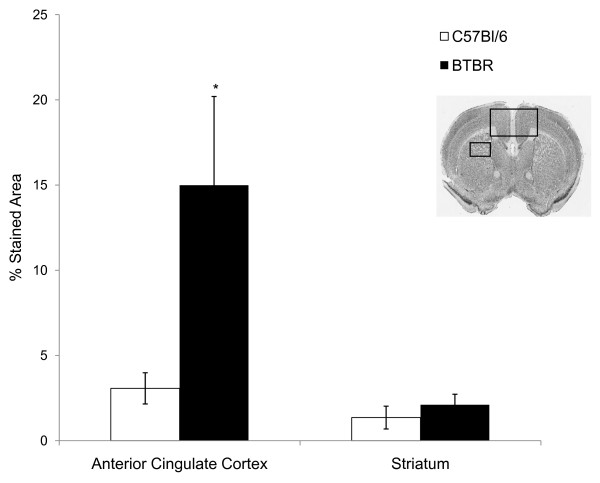
**Neuroglial proteoglycan NG2 immunoreactivity in the cingulate cortex is significantly increased in BTBR brain**. Quantitative image analysis of NG2 expression in the anterior cingulate cortex (ACC) of the BTBR mouse compared with the B6 mouse. Histogram illustrates measurement of percentage NG2 stained area from three sections per animal in the region of the ACC and the striatum. This resulted in a significant increase in the area of NG2 immunoreactivity in BTBR versus B6 mice. (*P = 0.0088, n = 6 mice per strain). No significant change in the expression of NG2 in the striatum was seen in the same animals. The coronal section illustrates the regions of interest chosen for image analysis: ACC (large rectangle) and striatum (small rectangle).

Polydendrocytes, the glial population defined by NG2, play a role in migration, neurogenesis, myelin repair and even modulation of synaptic activity [50,51]. An apparent increase in expression of NG2 immunoreactivity was seen in the anterior cingulate cortex (ACC) of BTBR forebrain (Figure 5) which spanned caudally into the retrosplenial cortex. Morphologic changes consisted of an increase in the staining intensity, size and extent of processes of NG2-positive cells in the BTBR mouse brain compared with controls (Figure 5). Quantitative image analysis of NG2 expression found a significant increase in the area of NG2 immunoreactivity in the cingulate cortex of BTBR mice compared with controls (*P *= 0.0088, n = 6 per strain) (Figure 6). By contrast, no significant differences in NG2 expression in the striatum from the same sections were seen (Figure 6), suggesting that NG2 increases were spatially restricted.

### Intact dendritic cytoarchitecture, mossy fibers and cholinergic fiber density are present in the BTBR brain

No specific alterations were seen in the overall organization and density of mossy-fiber synapses in the hippocampus as assessed by Timm staining (see Additional file 2, Figure S1D). Cholinergic fibers and neurons, as assessed by AchE histochemistry, had no specific alterations in BTBR versus B6 mouse forebrain (see Additional file 2, Figure S1B). To examine putative changes in the neuronal dendritic cytoskeleton, BTBR and B6 brains were evaluated with an antibody directed against microtubule associated protein MAP2. No specific changes were seen in MAP2 immunoreactivity in BTBR compared with control brain in any region (see Additional file 3, Figure S2).

### Synapse, excitatory and inhibitory markers are not profoundly altered in BTBR brain

To evaluate the overall structural integrity of synapses in the brains of BTBR and B6 mice, antibodies directed to specific presynaptic and postsynaptic structures were evaluated by immunohistochemistry. Evaluation of the two presynaptic markers synapsin 2 and synaptophysin, and of the postsynaptic markers postsynaptic density protein PSD95, drebrin and the glutamate receptor GluR1 AMPA receptor showed that there were no qualitative changes in BTBR compared with B6 mice in any forebrain region, with one exception, which was a focal disruption of synaptic staining in the region of the ectopic myelinated white-matter bundles present in the ACC (Figure 4), as expected in a region composed of white matter.

To examine potential alterations in excitatory and inhibitory systems, the distributions of GAD67, GAD65, parvalbumin (PVA) and vesicular glutamate transporter VGluT1 were evaluated. No qualitative changes were seen in VGluT1 expression throughout the forebrain, except for an absence of immunoreactivity in the ectopic white-matter regions of the cingulate cortex, in line with the specific reduction of synaptic markers in this same region (Figure 4). GAD65, GAD67 and PVA expression was found to be similar in both BTBR mice and B6 mice in all regions examined (data not shown).

### BTBR mice exhibit a significant reduction in adult hippocampal neurogenesis

The morphology of the hippocampus in BTBR mice appeared remarkably similar to that seen in homozygous *Emx1 *knockout mice, a model characterized by callosal agenesis [52] and defective neurogenesis [53]. This finding, as well as reports of augmented stress response in the BTBR model [54], led us to investigate the distribution of specific neural progenitors implicated in hippocampal neurogenesis.

Antibodies directed to immature neurons in the dentate gyrus SGZ were evaluated by examining the expression of PSA-NCAM and DCX, both recognized as markers of neurogenesis [55,56]. Marked reductions in the density of neurons immunoreactive for DCX and PSA-NCAM were seen throughout the hippocampal dentate gyrus of BTBR compared with B6 mice (see Additional file 1, Figure S3). Outside the hippocampus, DCX and PSA-NCAM immunoreactivities were expressed in cerebral sites of structural plasticity including the subventricular zone, the piriform and entorhinal cortices, and the amygdala, consistent with previous reports [57,58]. No qualitative changes in the expression of DCX and PSA-NCAM were seen in these brain areas in BTBR compared with B6 mice (data not shown). Immunohistochemistry for GAD67 and NG2 in the same region showed no specific changes in BTBR mice compared with controls (see Additional file 1, Figure S3). Quantitative image analysis in the dentate gyrus revealed a significant reduction in the expression of DCX in the BTBR compared with B6 mice (*P *< 0.05, n = 6 mice per strain) (see Additional file 1, Figure S3), whereas no changes in the oligodendrocyte precursor NG2 or the GABAergic marker GAD67 were seen in the SGZ (see Additional file 1, Figure S3). Such results suggest that the alterations are specific to neural and not glial progenitors. These results demonstrate the specificity of the alterations in DCX expression relative to other markers in the same region. The significant reduction of DCX expression in the hippocampal dentate gyrus in our study is consistent with decreased hippocampal neurogenesis [55,59].

**Figure 7:**
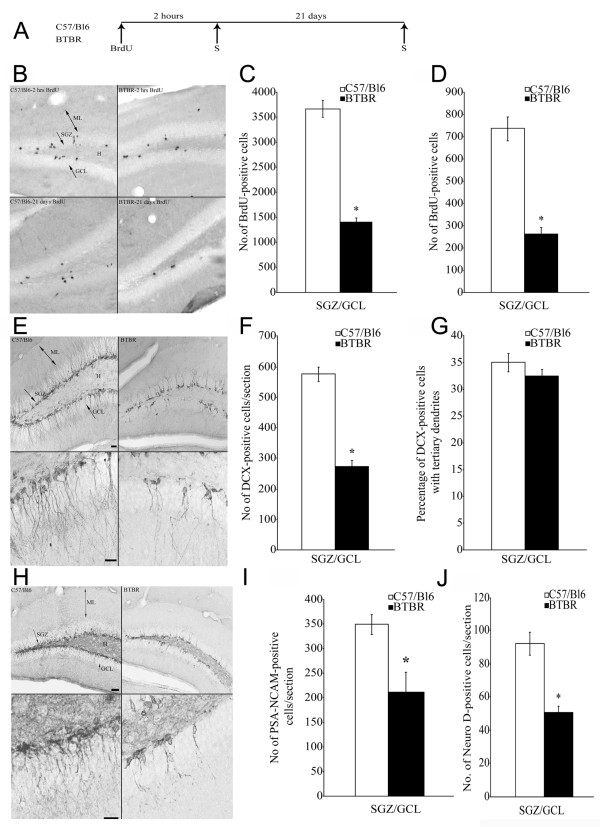
**Regulation of adult hippocampal neurogenesis in BTBR mice**. **(A) **Schematic representation of the experimental design to assess the regulation of adult hippocampal neurogenesis in BTBR mice (S time point represents time of euthanasia). Proliferation phase was assessed at 2 hours, and survival of neural precursors was examined at 21 days post bromodeoxyuridine (BrdU) administration. **(B) **Representative photomicrographs of BrdU-positive cells in the subgranular zone (SGZ)/granule-cell layer (GCL) of the B6 and BTBR animals. BrdU-positive cells were seen in the SGZ at the border of the GCL and hilus. **(C) **Quantitative unbiased stereological analysis revealed a significant decrease in the number of BrdU positive cells in the SGZ/GCL in the BTBR mice in the proliferation study. **(D) **Quantitative stereological analysis revealed a significant decrease in the BrdU-positive cells in the SGZ/GCL of the BTBR mice in the survival study. **(E) **Representative photomicrographs of DCX positive cells in the SGZ of the B6 and BTBR hippocampus, and the corresponding magnified image of the DCX positive cells with tertiary dendrites. **(F) **Analysis of DCX-positive cells per section in the SGZ/GCL region revealed a significant decrease in the BTBR animals compared with the B6. **(G) **Percentage of DCX positive cells with tertiary dendrites did not find any significant change between the B6 and BTBR animals. **(H) **Representative photomicrographs of cells positive for PSA-NCAM from B6 and BTBR animals, and the corresponding magnified images are shown. **(I) **Quantitative analysis of the PSA-NCAM positive cells in the BTBR compared with the B6 animals revealed a decreased number of PSA-NCAM positive cells per section in the BTBR animals. **(J) **Quantitative analysis revealed a significant decrease in the number of NeuroD positive cells per section in the SGZ/GCL of the BTBR compared with B6 animals. The results are expressed as mean ± SEM (n = 9/group). *P < 0.05 compared with the B6 animals (Student *t*-test). **(E, H) **Scale bars = 50 μm (top panel), 25 μm (bottom panel). H = hilus, ML = molecular layer.

To definitively address the hypothesis of alterations in adult hippocampal neurogenesis in the present study, an independent cohort of mice was administered BrdU, and assessed by unbiased stereological counting in the SGZ of the dentate gyrus. A significant decline in proliferating BrdU-positive progenitors was seen in the BTBR mice compared with controls (*P *< 0.05) (Figure 7B, C). The numbers of BrdU-positive cells persisting within the GCL of BTBR mice 21 days after BrdU treatment also had a similar significant reduction (*P *< 0.05) (Figure 7D). This decline in adult hippocampal neurogenesis revealed by the exogenous mitotic marker BrdU was also accompanied by a steep reduction in the numbers of immature neurons positive for DCX, PSA-NCAM and NeuroD within the hippocampal neurogenic niche of BTBR mice (*P *< 0.05) (Figure 7E, F, H, I, M). Whereas the numbers of DCX-positive cells were significantly reduced in BTBR mice, the extent of morphological maturation of DCX-positive immature neurons in BTBR mice did not differ compared with B6 controls, as shown by no change in the percentage distribution of DCX positive immature neurons bearing tertiary dendrites (Figure 7G). Further, we addressed whether the neuronal or glial differentiation of newborn progenitors within the hippocampal neurogenic niche was altered in BTBR mice. We saw a robust decline in the percentage of BrdU positive cells that colocalized with NeuN (*P *< 0.05) (see Additional file 4 Figure S4). BrdU positive cells were associated with a significant increase in astroglial proliferation, as shown by increased percentage colocalization with GFAP (*P *< 0.05) (see Additional file 4 Figure S4). Taken together, these results indicate that BTBR mice exhibit a robust reduction in adult hippocampal neurogenesis, with significant decreases in both progenitor proliferation and neuronal differentiation. Interestingly, those immature neurons that did seem to persist in BTBR dentate gyrus exhibited normal morphological maturation.

### BDNF mRNA is significantly reduced in BTBR hippocampus

**Figure 8:**
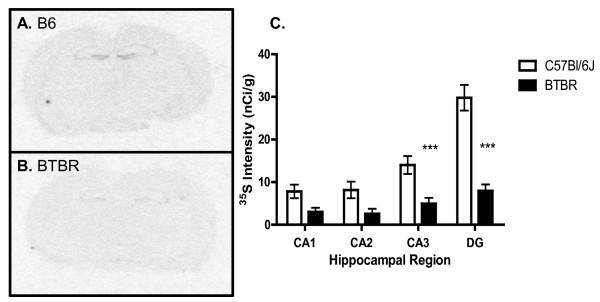
**Significant reduction of brain-derived neurotrophic factor (BDNF) mRNA in the BTBR hippocampus**. **(A) **There was prominent expression of BDNF mRNA in the hippocampus in B6 forebrain. **(B) **BDNF mRNA expression in the BTBR forebrain was markedly reduced compared with the B6 forebrain (compare (A) and (B)). **(C) **Quantitative densitrometry of the CA1, CA2, CA3 and dentate gyrus (DG) subregions of the hippocampus revealed that mRNA expression was significantly decreased in BTBR compared with B6 mice in the CA3 and dentate gyrus. The results are expressed as the mean ± SEM (n = 6/group). ***P ≤ 0.001 for both CA3 and DG, two-way ANOVA with Bonferroni *post hoc *analysis).

Given the central role of BDNF in modulating experience-dependent plasticity and its role in proliferation differentiation and survival of newly generated neurons [60], we examined BDNF gene expression in BTBR mice. *In situ *hybridization of BDNF mRNA revealed a significant reduction in the hippocampus of BTBR compared with B6 mice (Figure 8). BDNF transcripts were decreased by 73% in the dentate gyrus (mean ± SEM B6: 29 ± 3; BTBR: 14 ± 2; n = 6/group, *P *< 0.0001) and 65% in the CA3 region (B6: 14 ± 2.1; BTBR: 4.9 ± 1.4, n = 6/group, *P *< 0.001) with non-significant reductions in the CA1 and CA2 regions, suggesting spatial heterogeneity of BDNF changes.

Table 2 provides an inclusive summary of the results of the different cellular markers in BTBR compared with B6 mice.

**Table 2:** Results of histopathological changes in BTBR versus B6 mice

Marker	PFC	STR	ACC	AMG	HPC	Comments
AchE	0	0	0	0	0	
BrdU	N/A	N/A	N/A	N/A	-	
+ GFAP	N/A	N/A	N/A	N/A	+	
+ NeuN	N/A	N/A	N/A	N/A	-	
CNPase	0	0	+	0	0	Focal in CC region
Drebrin	0	0	-	0	0	
Doublecortin	0	0	0	0	-	
GAD65	0	0	-	0	0	
GAD67	0	0	-	0	0	
GFAP	0	0	0	0	0	Misorientation (alv., cing.)
GluR1	0	0	-	0	0	
Iba1	0	0	0	0	0	Misorientation (alv., cing.)
MAP2	0	0	0	0	0	
MBP	0	0	+	0	0	Focal in CC region
NeuroD	N/A	N/A	N/A	N/A	-	
NG2	0	0	++	0	0	
Parvalbumin	0	0	-	0	0	
PSA-NCAM	0	0	0	0	-	
PSD-95	0	0	-	0	0	
Synapsin 2	0	0	-	0	0	
Synaptophysin	0	0	-	0	0	
Timm's	0	0	0	0	0	
VGluT1	0	0	-	0	0	

PFC = prefrontal cortex; STR = striatum; ACC = anterior cingulate cortex; AMG = amygdala; HPC = hippocampus; AchE = acetylcholinesterase; BrdU = 5-bromo-2'-deoxyuridine; CNPase = 2',3'-cyclic nucleotide 3'-phosphodiesterase; GAD65 = glutamic acid decarboxylase 65 kDa isoform; GAD67 = glutamic acid decarboxylase 67 kDa isoform; GFAP = glial fibrillary acidic protein; GluR1 = glutamate receptor 1; Iba1 = ionized calcium-binding adaptor molecule 1; MAP2 = microtubule-associated protein 2; MBP = myelin basic protein; N/A: not analyzed; NeuN = neuronal nuclei; PSA-NCAM = poly-sialic acid neural cell adhesion molecule; PSD-95 = postsynaptic density protein of 95 kDa; VGluT1 = vesicular glutamate transporter 1.

Comments refer to alterations probably related to callosal agenesis in the BTBR mice (-) = decreased staining compared with B6; (0) = equal staining compared with B6; (+) = moderately increased staining compared with B6; (++) = strongly increased staining compared with B6.

## Discussion

We report extensive histopathological characterization of the brain of the BTBR inbred mouse strain, and describe novel changes in specific markers within key forebrain regions. Specifically, a significant increase in the expression of the oligodendrocyte precursor NG2 in the ACC and marked reductions in the number of neural precursors positive for DCX, PSA-NCAM and NeuroD in the hippocampus were seen. Despite the presence of complete callosal agenesis, surprisingly few changes in the majority of markers were found in most brain regions (Table 2). Given the number of neuronal-, glial-, synaptic- and neurotransmitter-related markers we examined, the modest extent of global changes in neurostructural proteins in response to such a marked perturbation of normal brain development is striking. No evidence of structural or antigenic changes was seen in most brain regions using markers such as MAP2 for neuronal dendrites, Timm staining for mossy fibers, and AchE histochemistry for cholinergic pathways, and no specific changes in the expression of excitatory (VGluT1) or inhibitory (GAD67, GAD65, PVA) markers were seen. Such results do not exclude the possibility that subtle changes might be found using higher-resolution approaches such as electron microscopy. Moreover, non-histologic assays such as neurochemistry and electrophysiology represent complimentary mechanistic approaches for addressing the contribution of functional deficits to the autism-like behaviors seen in the BTBR mouse.

Our intent was to profile a panel of diverse cellular and molecular markers in the BTBR mouse as opposed to addressing one or a few specific mechanistic hypotheses. Given that the BTBR model is proposed to mimic the human behavioral impairments of autism, the relevance of our findings to human neuropathology of autism is worth consideration. The current understanding of the neuropathology of human idiopathic autism is based on relatively limited numbers of cases. The most consistent findings reported to date include defective cortical minicolumns [61], reduction in neuron number and size in key brain areas, and increased dendritic spine density [38,62-66]. Additional findings include loss of Purkinje cells [67], alterations in specific GABAergic receptors [68,69], changes in markers of the cholinergic system [70,71], focal increases in interneurons [72], and increased glial activation [73-75]. Based on this information, some comparisons with our present findings include lack of changes in brain weight, and absence of changes in cholinergic fiber density and PVA-expressing interneurons in BTBR brain, at least as analyzed qualitatively. These results suggest that differences exist between the pathology of BTBR and human autism. Another difference is that we found no evidence of glial activation in the BTBR brain. The gliosis seen in postmortem examination of human autism cases might have arisen from environmental, inflammatory (both of which have been implicated in autism) or other epigenetic mechanisms that are not present in the mouse model. Additionally, seizures, which occur in up to 30% of patients with ASD [76,77], or concomitant drug therapy can alter glial morphology or activation [78,79]. These confounding factors cannot be controlled for in human postmortem studies, and pose particular challenges in ASD, for which a paucity of human postmortem brain tissue is available. Such factors re-emphasize the need for relevant translational animal models for ASD. Future studies using more comprehensive evaluations such as spine morphology, minicolumn assessments and receptor autoradiography represent suitable techniques to compare the neuropathology of the BTBR mouse more directly with postmortem human autistic brain. Indeed, receptor autoradiography in BTBR mice has revealed neurochemical changes in the serotonergic system [22] that are consistent with alterations in serotonergic receptor systems in human autistic brain [80].

In +the present study, we defined specific cellular and anatomic changes in glia, a population of cells that would be expected to change as a consequence of white matter defects. There was reduced expression of the myelin markers MBP and CNPase in midline forebrain structures, findings that would be predicted from reduced white matter content and callosal agenesis. Likewise, misorientation of glial fibers in the alveus and cingulum are probably a consequence of callosal agenesis, given that it is known that existing fibers can be rerouted in different orientations. Alterations in CNPase and MBP in focal regions of the cortex may be related to the presence of Probst bundles, which have been defined as aberrant, longitudinally oriented, white matter bundles near the midline that are believed to be composed of rerouted callosal fibers [27]. Probst bundles have been previously described in BTBR mice [20] and in other mouse models of callosal agenesis (for example, netrin1 [81], NF1a [82], ddN [83], RI-I [84], Emx-1 [52]) and in human patients with partial agenesis of the corpus callosum [85]. The relationship of the small ectopic white-matter bundles in the ACC to Probst bundles is unclear, and additional experiments, such as fiber tract tracing experiments, which were historically used to define Probst bundles [86,87], may be illuminating. Fiber tracing experiments may also help characterize the misoriented glial fibers in the alveus and cingulum. CNPase and MBP immunoreactive areas in regions of ectopic white matter bundles were devoid of the synaptic antigens synaptophysin, PSD95, VGluT1, synapsin 2 and drebrin. This is expected, given that the ectopic structures are composed of white matter. However, it is noteworthy that disruption of synaptic cytoarchitecture and integrity in the ACC may lead to significant functional consequences for higher-order functions such as attention, executive function, and integration of emotion and cognitive processes. In autism, abnormalities in the ACC have been identified using neuroimaging methods [88-91]. Notably, the ACC is reported to display anatomic and functional alterations in several human neuropsychiatric conditions, including schizophrenia and bipolar disease, in addition to autism [92-95]. Translational research of higher order functional circuits using endpoints such as neuroimaging and electroencephalography in both mouse and human callosal agenesis/dysgenesis and in patients with ASD may address key hypotheses about the relationship between ACC alterations and neuropsychiatric conditions.

When comparing the relative changes of all the markers evaluated in our study, the most robust changes were seen for DCX, PSA-NCAM, BDNF and NG2 expression. It is of particular interest that these markers are known to play a role in neuronal development and plasticity. Selective changes in such a panel of neurodevelopmental proteins are consistent with a congenital defect in neurodevelopment. The chondroitin sulfate proteoglycan NG2 is known to regulate cell proliferation and motility ([96]), modulate axon growth [97], and prevent axon regeneration [98]. NG2 cells in both white and gray matter engage not only in the genesis of oligodendrocytes during development, but also in remyelination of demyelinated axons in the adult nervous system [99]. NG2 cells form synaptic junctions with axons, and participate in glutamatergic [100,101] and GABAergic [102] signaling with neurons. Very little is known about the changes in oligodendrocyte precursors in disease states, with the exception of multiple sclerosis, in which NG2 has been suggested to prevent remyelination [103]. Whether the increased expression of NG2-positive cells represents an attempt to remyelinate the absent corpus callosum, or may function to participate in defective neuron-glia communication is unknown. Future experiments aimed at quantifying the number of NG2 positive cells and the changes in NG2 positive processes may further elucidate the mechanisms of specific alterations in polydendrocytes in this and other models of callosal anomalies. Expression of these specific neuronal and glial progenitors in human postmortem brain from cases of autism or callosal agenesis has not been reported to date. This finding represents an important area for future investigation in human postmortem brain, and is an example of how the BTBR mouse model has the potential to uncover novel neuroanatomical features of human disease.

The relationship between callosal anomalies and behavioral impairment in mice is complex. Several studies have examined the behavioral phenotypes of mouse models of callosal abnormalities; interestingly, very mild phenotypes have been reported across a wide range of basic behavioral assays [23]. Many different mouse models exhibit callosal abnormalities [104], yet only very few reportedly exhibit behavioral deficits resembling autism [18], indicating that the relationship, as in humans, is not a simple one. The BALB/c mouse is one example other than the BTBR mouse that exhibits social deficits and callosal abnormalities [1,23,105], yet other models of callosal dysgenesis (such as the J1 strain, which is phylogenetically similar to BTBR) do not exhibit impaired social interactions. To address a putative relationship more directly, Yang *et al*. [20] reported that surgical transection of the corpus callosum in B6 mice at postnatal day 7 did not result in the same autism-like behavioral deficits seen in the BTBR model, leading the authors to suggest that callosal abnormalities are not responsible for the behavioral deficits in autism. Similarly, commissurotomy in humans, also termed split-brain or disconnection syndrome, does not result in the same behavioral abnormalities as in cases of callosal agenesis [27,106]. Clearly, further studies in animals and in humans are required to directly address the hypothesis that callosal abnormalities contribute to the behavioral deficits of autism.

The most profound changes in the present study occurred in the hippocampus. The hippocampus plays an important role in memory functions, emotional behavior, processing of novel information and integrating social information, all domains affected in ASD. Several relevant genetic mouse models of neuropsychiatric disorders emphasize the potential role of reduced neurogenesis on autism-related behaviors, including chromosome 22q11 deletion [13], mutant *Disc1 *transgenic mice [24] and *Reelin *knockout mice [107]. Moreover, reduced hippocampal neurogenesis has been reported in the *Emx1 *knockout mouse, a model that also exhibits callosal agenesis [53]. Intriguingly, *Disc1 *truncation produces callosal abnormalities in mice [24], and *Disc1 *single nucleotide polymorphisms are associated with autism, with the strongest association occurring in males [108]. Reduced hippocampal neurogenesis is associated with stress, depression and defects in cognitive function [109]; the observation that stress abnormalities have been reported in BTBR mice [34] suggests a putative link.

Reduction in BDNF mRNA in the hippocampus is consistent with the reduction in neurogenesis. Hippocampal BDNF mRNA levels were most dramatically reduced in the dentate gyrus, with less significant reductions noted in the CA1 region. Recently, Silverman *et al*. reported reduced levels of BDNF protein in BTBR compared with B6 hippocampus, using biochemical methods [21], consistent with the present study. These results are also consistent with findings in other models of stress in which reduced neurogenesis is accompanied by changes in BDNF; however, the magnitude of reductions in the BTBR model in our studies is more profound than has been reported under conditions of stress [110-112]. Future experiments evaluating the effects of therapies that regulate neurogenesis and/or BDNF levels represent opportunities to decipher the role of these changes in reversing behavioral abnormalities. Therapeutic targets such as histamine H3 [113] and 5-hydroxytryptamine 5-HT6 receptor selective antagonists [114] and AMPA receptor modulators [21] represent good examples. With respect to relevance to human autism, patients with ASD have volumetric and structural changes in the hippocampus as revealed by neuroimaging approaches [115,116]. The recent postmortem study illustrating that impairments in neurogenesis may occur in some cases of human autism [117] suggests the potential translational relevance of at least one neuropathology finding in the present study.

## Conclusions

Neuropathological studies of the BTBR strain and callosal agenesis/dysgenesis models have in general been rather limited. The present findings extend the previous gross anatomic findings to defining more specific cellular and molecular changes in BTBR mice. Such findings direct investigation of key hypotheses directed at linking anatomic changes to functional deficits, and have potential implications for developing effective treatments for autism. Translational research is a crucial success factor for developing successful drugs into market, and is particularly imperative in nervous system diseases, for which the advancement of therapeutic candidates into humans is particularly challenging [118]. Construct validity is particularly important in cases for which no clinically effective medications exist and there is a lack of sufficient information about the etiology of the disease [119], which is indeed the case for ASD. The finding that the BTBR model recapitulates the three core deficits of human ASD is important, as this is a disease that is diagnosed based on purely behavioral outcomes. Given that behavioral interventions and pharmacotherapies both have been shown to reverse at least one of the core deficits in the model [6,19,20,22], some construct validity of the BTBR model to human autism is possible. The neuroanatomic characteristics of the BTBR model identify pathogenic neural substrates that may underlie behavioral deficits, and further suggest new potential therapeutic targets or strategies for intervention in autism.

## Competing interests

Eight (DS, SO, EA, HS, RR, BC, MP, DM) of the twelve authors are employees of Pfizer Global Research and Development, which funded this study at the time of submission of the manuscript.

## Authors' contributions

DS designed and directed the studies, interpreted data and wrote the manuscript. SO performed and coordinated all histopathology studies, procured samples and tissues, prepared figures and tables, and contributed to data interpretation and preparation of the manuscript. DM evaluated the pathology results of most of the markers used in the study, interpreted results and assisted in preparation of the paper. HS and SO performed the Aperio image analysis, and cut and stained the mossy-fiber sections for specific markers. FD carried out all the free-floating experiments and special stains. MP, BC and RR contributed intellectually to the design and interpretation of the results, and the editing of the manuscript. SS and AT performed the staining and stereology experiments for the neurogenesis measures. VV contributed intellectually to the design and interpretation of the neurogenesis experiments and contributed to the manuscript. The BDNF experiments and data analysis were performed by EA. All authors reviewed the manuscript before submission.

## Supplementary Material

Additional file 1 **Figure S3 Selective reduction of doublecortin (DCX) immunoreactivity in BTBR hippocampus**. **(A, B) **DCX immunoreactivity in the dentate gyrus (DG) of **(A) **BTBR and **(B) **B6 forebrain. Marked reduction in DCX-positive neural progenitors was seen in BTBR DG (compare **A **with **B**). Specific changes included reduced frequency of DCX-immunoreactive neurons in the SGZ. **(C) **Significant reduction in DCX expression in the BTBR DG by quantitative image analysis (*P = 0.024, n = 6 per strain). **(D, E) **Immunolocalization of neuroglial proteoglycan NG2 in the hippocampal dentate gyrus (DG) in representative (**D**) BTBR and **(E) **B6 brains. NG2 immunoreactivity was visible as polydendrocyte cell bodies and processes throughout the hilus and DCX-positive layers. **(F) **No significant differences in NG2 between BTBR and B6 DG were measured by quantitative image analysis. **(G, H) **Glutamic acid decarboxylase GAD67 immunoreactivity was visible in cell bodies and fibers and terminals in the hilus, subgranular zone (SGZ) and DCX positive layer (GCL) of the DG. **(I) **No specific qualitative changes in GAD67 immunoreactivity were seen in BTBR compared with B6 hippocampus. The free floating sections stained with each antibody are nearly adjacent to one another. Scale bar = 50 μm. Click here for file

Additional file 2 **Figure S1 No overt changes in cholinergic and mossy fibers are present in BTBR compared with B6 mouse forebrain**. Representative sections of **(A, C) **BTBR and **(B, D) **B6 mouse forebrain stained with **(A, B) **acetylcholinesterase (AchE) histochemistry or **(C, D) **Timm stain. No obvious qualitative differences between BTBR and B6 sections were seen at any magnification. Scale bar = 1000 μm. Click here for file

Additional file 3 **Figure S2 No overt changes in dendritic cytoarchitecture are present in BTBR compared with B6 mouse forebrain**. Representative sections of **(A, C) **BTBR and **(B, D) **B6 mouse forebrain stained with microtubule-associated protein MAP2 at the level of the dorsal hippocampus. (**C, D**) MAP2 immunoreactivity in the CA1 region of the hippocampus. No obvious differences between BTBR and B6 sections were seen. Scale bars *= ***(A, B) **1000 μm; **(C, D) **100 μm. Click here for file

Additional file 4 **Figure S4 Dual localization of 5-bromo-2'-deoxyuridine (BrdU) with neuronal or glial markers in the SGZ**. **A, B**: Representative confocal z-stack images of a BrdU-positive cell colocalized with **(A) **neuronal nuclei (Neu N) or **(B) **glial fibrillary acidic protein (GFAP), respectively. **(C) **Confocal z-stack analysis of the BrdU-positive cells indicated that the percentage colocalization of the BrdU-positive cells with the neuronal marker; NeuN was significantly reduced and the astrocytic marker GFAP was significantly increased. The results are expressed as the mean ± SEM (n = 9/group). *P < 0.05 compared with the B6 animals (Student *t*-test). Click here for file
