# Supplementary figures and images for: Histopathologic characterization of the BTBR mouse model of autistic-like behavior reveals selective changes in neurodevelopmental proteins and adult hippocampal neurogenesis
Source: Mol Autism. 2011 May 16;2:7. doi: 10.1186/2040-2392-2-7 (PMC3135520; doi:10.1186/2040-2392-2-7)

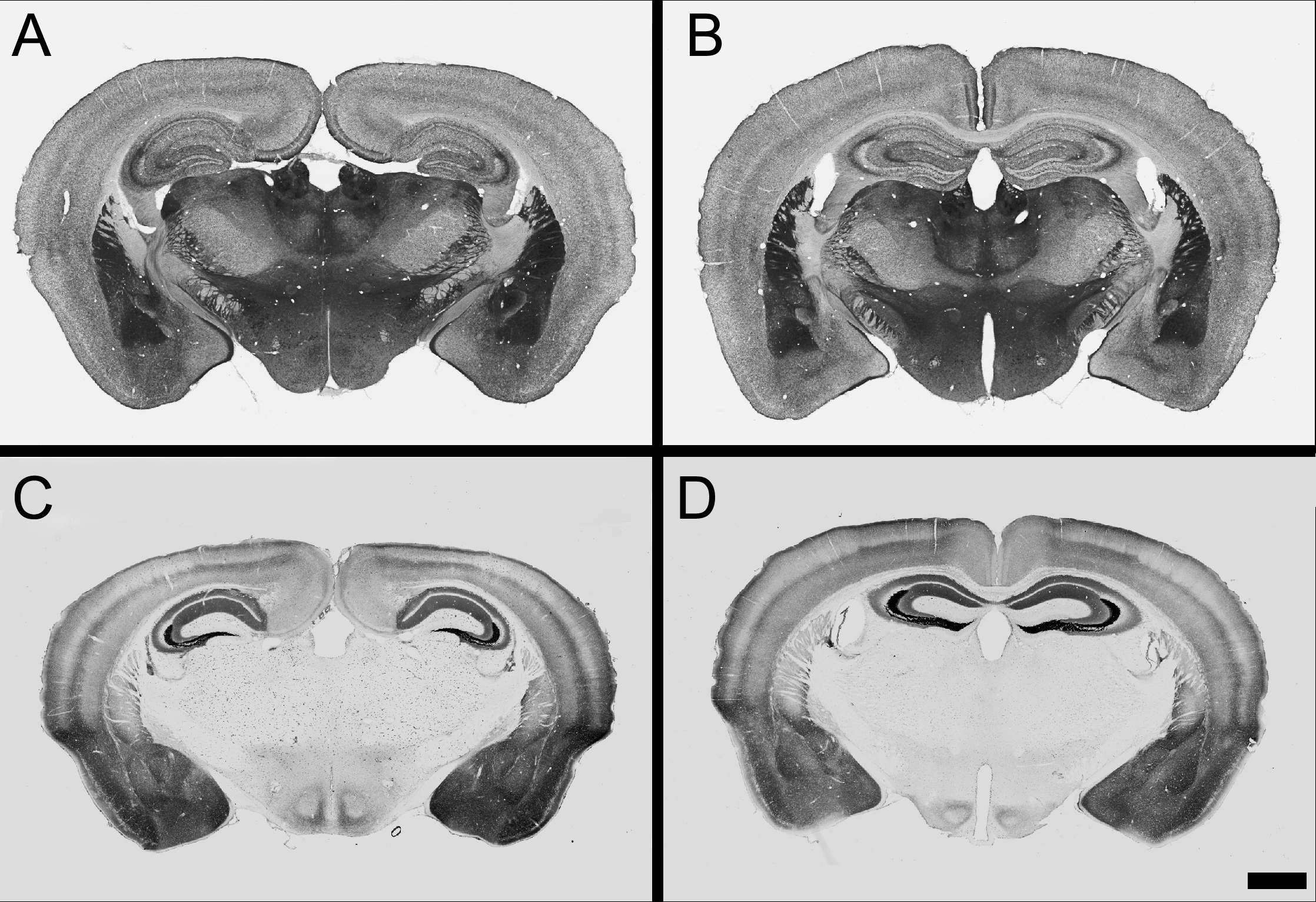

Supplement: Additional file 1 — Figure S3 Selective reduction of doublecortin (DCX) immunoreactivity in BTBR hippocampus. (A, B) DCX immunoreactivity in the dentate gyrus (DG) of (A) BTBR and (B) B6 forebrain. Marked reduction in DCX-positive neural progenitors was seen in BTBR DG (compare A with B). Specific changes included reduced frequency of DCX-immunoreactive neurons in the SGZ. (C) Significant reduction in DCX expression in the BTBR DG by quantitative image analysis (*P = 0.024, n = 6 per strain). (D, E) Immunolocalization of neuroglial proteoglycan NG2 in the hippocampal dentate gyrus (DG) in representative (D) BTBR and (E) B6 brains. NG2 immunoreactivity was visible as polydendrocyte cell bodies and processes throughout the hilus and DCX-positive layers. (F) No significant differences in NG2 between BTBR and B6 DG were measured by quantitative image analysis. (G, H) Glutamic acid decarboxylase GAD67 immunoreactivity was visible in cell bodies and fibers and terminals in the hilus, subgranular zone (SGZ) and DCX positive layer (GCL) of the DG. (I) No specific qualitative changes in GAD67 immunoreactivity were seen in BTBR compared with B6 hippocampus. The free floating sections stained with each antibody are nearly adjacent to one another. Scale bar = 50 μm. [file 2040-2392-2-7-S1.TIFF]

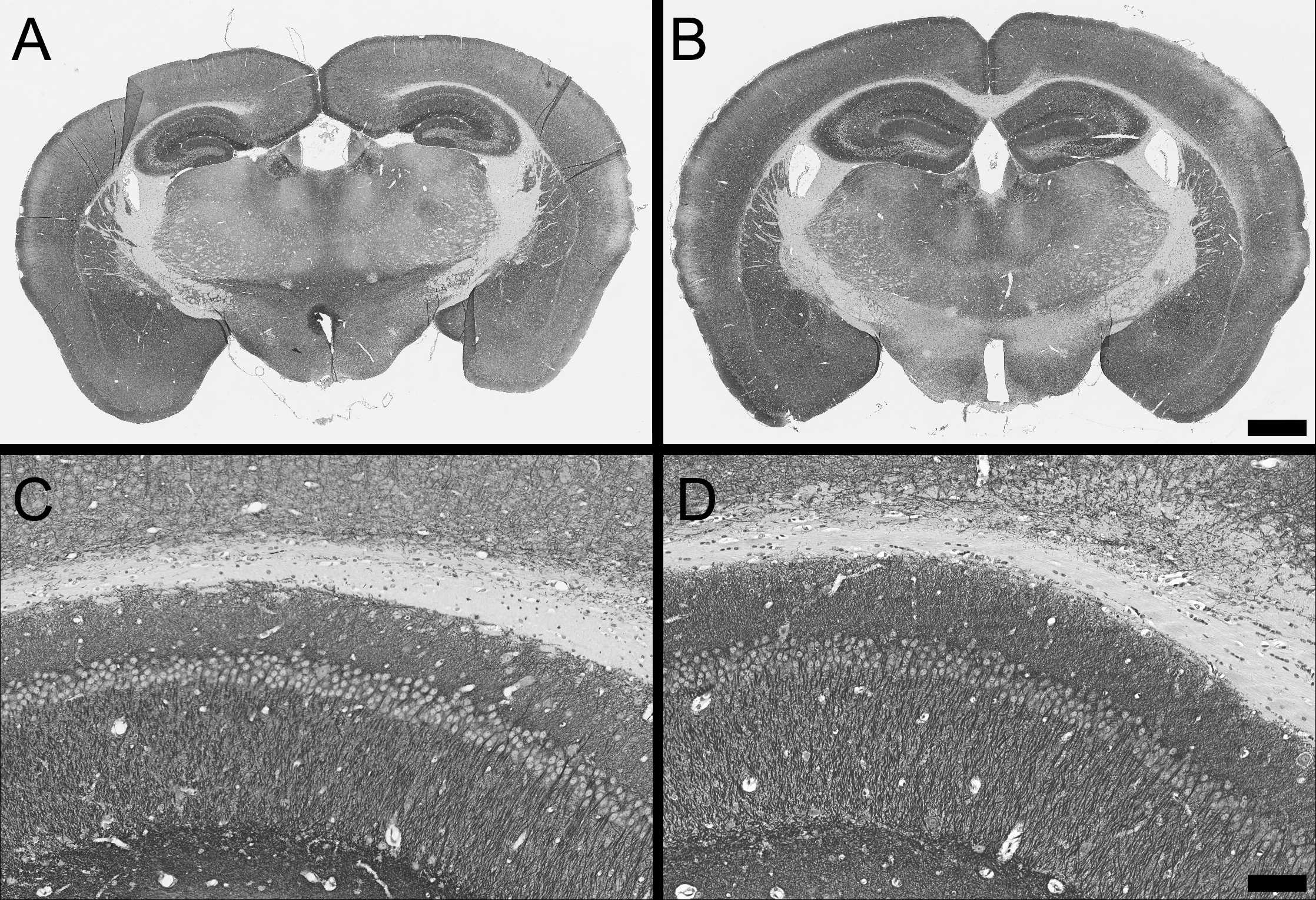

Supplement: Additional file 2 — Figure S1 No overt changes in cholinergic and mossy fibers are present in BTBR compared with B6 mouse forebrain. Representative sections of (A, C) BTBR and (B, D) B6 mouse forebrain stained with (A, B) acetylcholinesterase (AchE) histochemistry or (C, D) Timm stain. No obvious qualitative differences between BTBR and B6 sections were seen at any magnification. Scale bar = 1000 μm. [file 2040-2392-2-7-S2.TIFF]

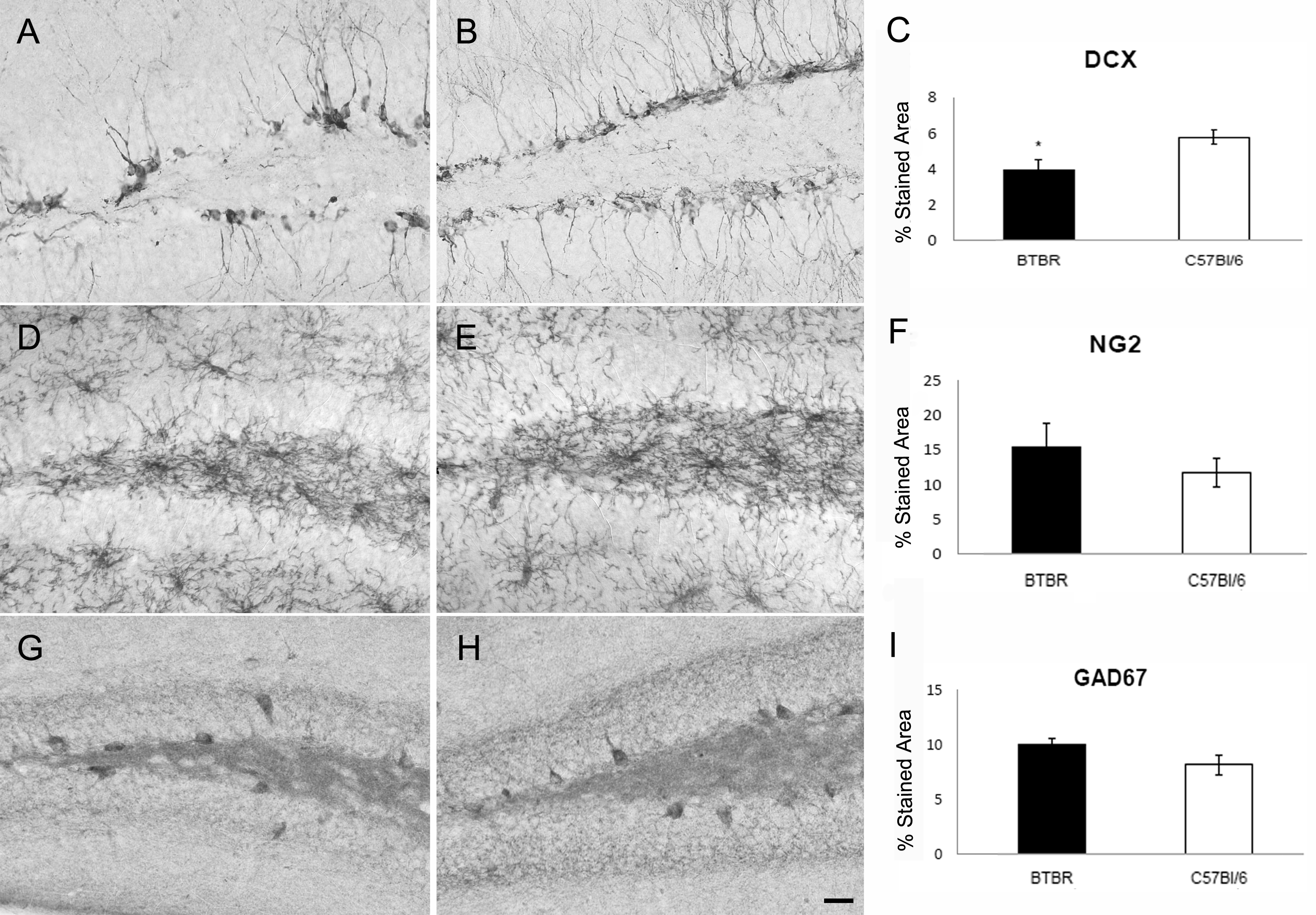

Supplement: Additional file 3 — Figure S2 No overt changes in dendritic cytoarchitecture are present in BTBR compared with B6 mouse forebrain. Representative sections of (A, C) BTBR and (B, D) B6 mouse forebrain stained with microtubule-associated protein MAP2 at the level of the dorsal hippocampus. (C, D) MAP2 immunoreactivity in the CA1 region of the hippocampus. No obvious differences between BTBR and B6 sections were seen. Scale bars = (A, B) 1000 μm; (C, D) 100 μm. [file 2040-2392-2-7-S3.TIFF]

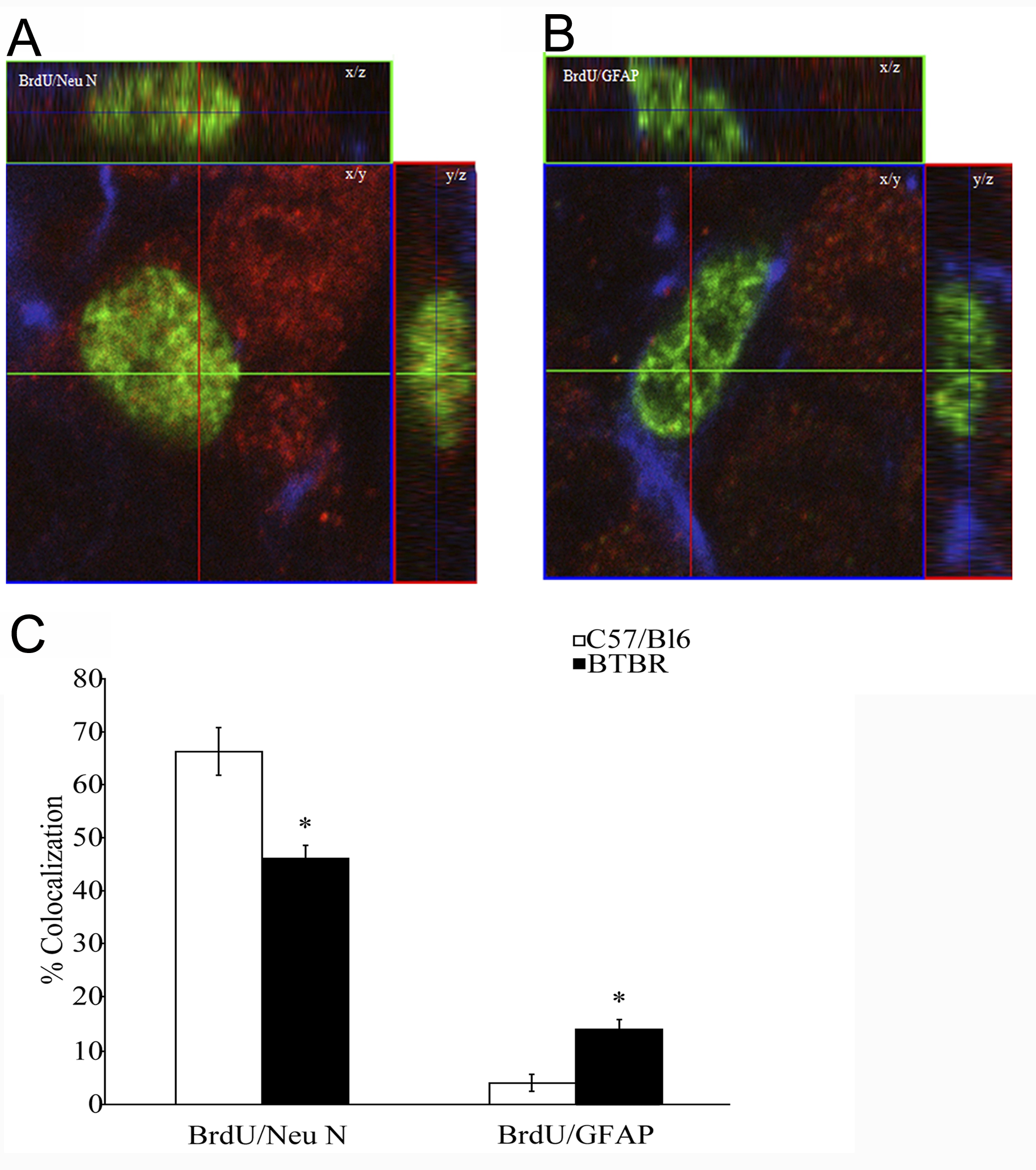

Supplement: Additional file 4 — Figure S4 Dual localization of 5-bromo-2'-deoxyuridine (BrdU) with neuronal or glial markers in the SGZ. A, B: Representative confocal z-stack images of a BrdU-positive cell colocalized with (A) neuronal nuclei (Neu N) or (B) glial fibrillary acidic protein (GFAP), respectively. (C) Confocal z-stack analysis of the BrdU-positive cells indicated that the percentage colocalization of the BrdU-positive cells with the neuronal marker; NeuN was significantly reduced and the astrocytic marker GFAP was significantly increased. The results are expressed as the mean ± SEM (n = 9/group). *P < 0.05 compared with the B6 animals (Student t-test). [file 2040-2392-2-7-S4.TIFF]
